# Mobility Enhancement in Amorphous In-Ga-Zn-O Thin-Film Transistor by Induced Metallic in Nanoparticles and Cu Electrodes

**DOI:** 10.3390/nano8040197

**Published:** 2018-03-27

**Authors:** Shiben Hu, Honglong Ning, Kuankuan Lu, Zhiqiang Fang, Yuzhi Li, Rihui Yao, Miao Xu, Lei Wang, Junbiao Peng, Xubing Lu

**Affiliations:** 1 Institute of Polymer Optoelectronic Materials and Devices, State Key Laboratory of Luminescent Materials and Devices, South China University of Technology, Guangzhou 510640, China; hushiben@foxmail.com (S.H.); kk-lu@foxmail.com (K.L.); fangzq1230@126.com (Z.F.); liyuzhi1991@foxmail.com (Y.L.); xumiao4049@126.com (M.X.); mslwang@scut.edu.cn (L.W.); psjbpeng@scut.edu.cn (J.P.); 2 Institute for Advanced Materials, Guangdong Provincial Key Laboratory of Quantum Engineering and Quantum Materials, South China Normal University, Guangzhou 510006, China; luxubing@m.scnu.edu.cn

**Keywords:** IGZO, thin film transistors, indium, copper, Schottky contact

## Abstract

In this work, we fabricated a high-mobility amorphous indium-gallium-zinc-oxide (a-IGZO) thin-film transistor (TFT) based on alumina oxide (Al${}_{2}$O${}_{3}$) passivation layer (PVL) and copper (Cu) source/drain electrodes (S/D). The mechanism of the high mobility for a-IGZO TFT was proposed and experimentally demonstrated. The conductivity of the channel layer was significantly improved due to the formation of metallic In nanoparticles on the back channel during Al${}_{2}$O${}_{3}$ PVL sputtering. In addition, Ar atmosphere annealing induced the Schottky contact formation between the Cu S/D and the channel layer caused by Cu diffusion. In conjunction with high conductivity channel and Schottky contact, the a-IGZO TFT based on Cu S/D and Al${}_{2}$O${}_{3}$ PVL exhibited remarkable mobility of 33.5–220.1 cm${}^{2}$/Vs when channel length varies from 60 to 560 μm. This work presents a feasible way to implement high mobility and Cu electrodes in a-IGZO TFT, simultaneously.

## 1. Introduction

As display technology advances in terms of size, resolution and refresh rate, a high mobility thin film transistor (TFT) array with low resistivity interconnection lines is required to decrease resistance–capacitance delay to avoid signal distortions [1,2]. Amorphous oxide semiconductor (AOS) TFTs have attracted considerable attention on the flat panel display backplanes because of their high mobility and excellent uniformity [3]. On the other hand, a low resistivity metal line is required to meet the stringent demand for high resolution (≥UHD), large panel size (≥80 inch) and high refresh rate (≥240 Hz) [4,5,6]. In this regard, copper (Cu) is an appropriate electrode material for its lower resistivity than aluminum (Al) [7,8]. However, Cu atoms tend to diffuse into metal oxide semiconductors as acceptors or acceptor-like traps [9,10] during thermal annealing, resulting in degradation of electrical properties. This effect limits its application in TFT array backplanes as source/drain electrodes (S/D) that involve direct contact with AOS.

However, in this paper, Cu S/D play an important role in the performance of amorphous indium-gallium-zinc oxide (a-IGZO) TFTs. In one aspect, Al${}_{2}$O${}_{3}$ passivation layer (PVL)-induced metallic indium nanoparticles significantly improved the conductivity of the a-IGZO channel layer. On the other hand, Cu S/D formed Schottky contact with the channel layer after being annealed in an Ar atmosphere, which was advantageous for the a-IGZO TFT to achieve typical switching characteristics. Through these two effects, the a-IGZO TFT achieved mobility of up to 220.7 cm${}^{2}$/Vs.

## 2. Results and Discussion

**Figure 1:**
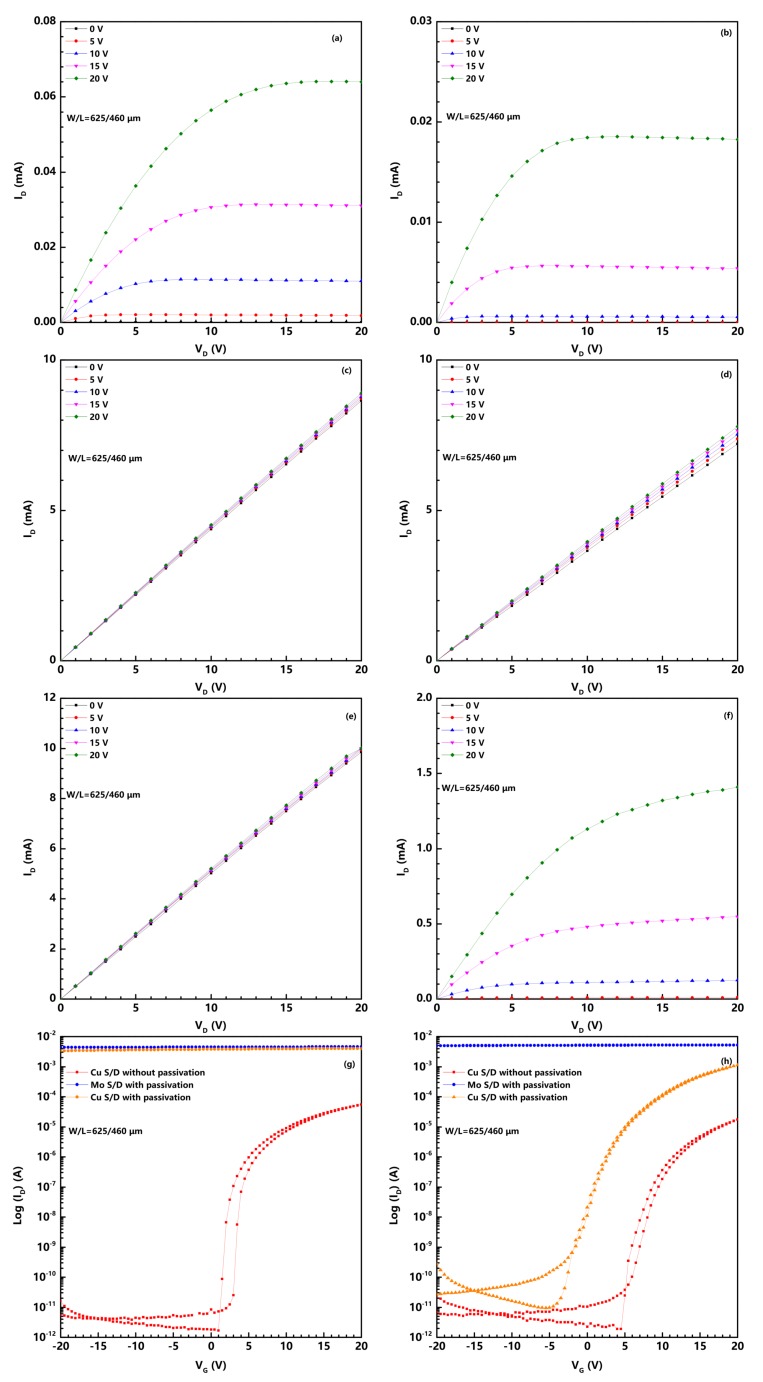
(**a**–**f**) output characteristics for S1, S2, S3, S4, S5 and S6 devices, respectively; transfer curves of a-IGZO TFTs (**g**) before post-annealing and (**h**) after post-annealing measured at drain voltage ($V_{D}$) equal to 10.1 V.

We prepared three kinds of a-IGZO TFTs, including non-passivated Cu-contact device, passivated Mo-contact device and passivated Cu-contact device. Before the post-annealing process, the three devices were labeled as S1, S3, and S4. After the post-annealing process, they were marked as S2, S5, and S6. Figure 1 shows the representatives’ output and transfer curves of the above six TFTs. Moreover, the electrical performance of the six TFTs is summarized in Table 1. In this case, the field effect mobility (μ${}_{FE}$) and the threshold voltage (*V*${}_{th}$) were extracted through the curve of the square root of the drain current (*I${}_{D}^{1/2}$*) versus the gate voltage (*V*${}_{G}$) in saturated operation region using the following equation:

$$I_{D}^{1/2}={\left(\frac{WC_{i}\mu_{FE}}{2L}\right)}^{1/2}\left(V_{G}-V_{th}\right).$$

Here, *W* and *L* are channel width and length, respectively. *C*${}_{i}$ is the gate capacitance per unit area of the insulator layer. Moreover, the subthreshold swing (*SS*) was extracted as the inverse of the maximum slope of the curve of Log *I*${}_{D}$ versus the *V*${}_{G}$ [11]:

$$SS={\left[{\left(\frac{dLogI_{D}}{dV_{G}}\right)}_{max}\right]}^{-1}.$$

The S2 device that suffered post-annealing treatment exhibited a degraded performance compared with the S1 device. It is a common phenomenon in many reports due to the diffusion of Cu caused by the thermal treatment [12]. It is obvious that the drain current of the S3–S5 devices could not be modulated by gate voltage (*V*${}_{G}$). These samples showed conductor behavior with an almost constant current versus gate voltage swept. The maximum drain current of the S3–S6 devices significantly increased compared with the S1 and S2 devices that have no Al${}_{2}$O${}_{3}$ PVL. This phenomenon indicates a high conductivity a-IGZO channel formed by the deposition of Al${}_{2}$O${}_{3}$ PVL. Compared to the S1–S5 devices, the S6 device showed a μ${}_{FE}$ of 220.7 cm${}^{2}$/Vs, a *V*${}_{th}$ of 5.7 V, an SS of 0.78 V/dec and an *I*${}_{on}$/*I*${}_{off}$ of 1.1 × 10${}^{8}$. Therefore, we can attribute the high performance of the S6 device to the Al${}_{2}$O${}_{3}$ PVL and Cu S/D.

**Figure 2:**
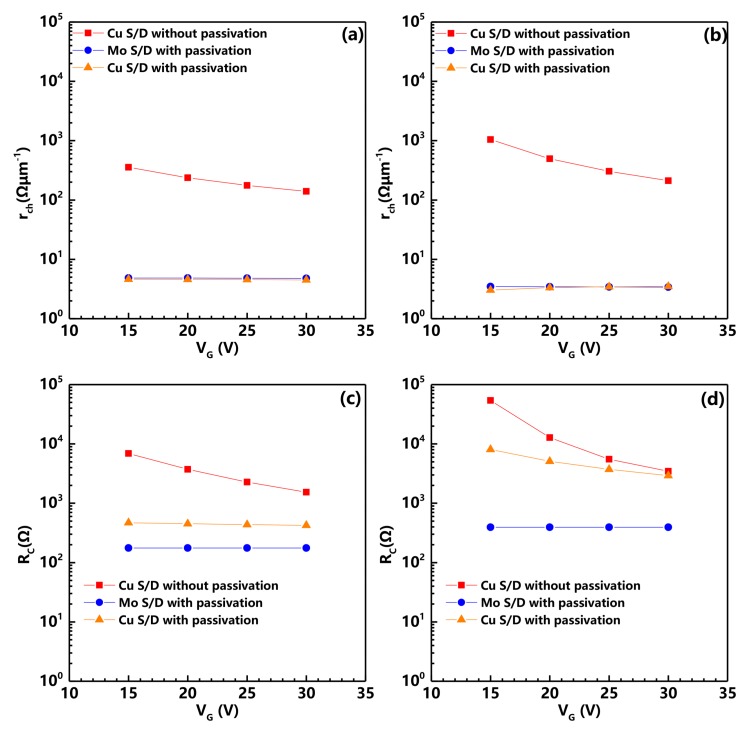
Channel resistance per unit channel length ($r_{ch}$) for a-IGZO TFTs (**a**) before post-annealing and (**b**) after post-annealing, and contact resistance ($R_{C}$) for a-IGZO TFTs (**c**) before post-annealing and (**d**) after post-annealing as a function of $V_{G}$.

For evaluating the effect of Al${}_{2}$O${}_{3}$ PVL and Cu S/D, a transmission line method (TLM) [13] was utilized to trace the variation of the channel and contact resistance using the TFT with different channel length. The total resistance (*R*${}_{T}$) was defined as the sum of channel resistance and contact resistance by the following equation at $V_{D}$ = 0.1 V:

$$R_{T}=\frac{V_{D}}{I_{D}}=r_{ch}L+R_{C},$$

where *r*${}_{ch}$ is the channel resistance per unit channel length, and $R_{C}$ is the total contact resistance. Figure 2 exhibits the variation of $R_{C}$ and $r_{ch}$ versus gate voltage before and after the post-annealing process. It is observed that the $r_{ch}$ of the S3–S6 devices that were passivated by Al${}_{2}$O${}_{3}$ films significantly decreased compared with the S1 and S2 devices. This phenomenon indicates that the sputtering of Al${}_{2}$O${}_{3}$ PVL can dramatically reduce the channel resistance in a-IGZO TFTs. For contact resistance evolution, the $R_{C}$ of the S2 and S6 devices significantly increased after post-annealed treatment. This increase is consistent with the previous report that Cu diffusion leads to a decrease in carrier concentration at the contact area [14]. Finally, we found that the S5 device has the similar $r_{ch}$ and lower $R_{C}$ as the S6 device. However, the S5 device behaved like a conductor, and the S6 device exhibited typical switching characteristics. According to their electrical properties, we can infer that high contact resistance caused by Cu diffusion and low channel resistance due to Al${}_{2}$O${}_{3}$ sputtering should be responsible for the high performance of the S6 device.

**Figure 3:**
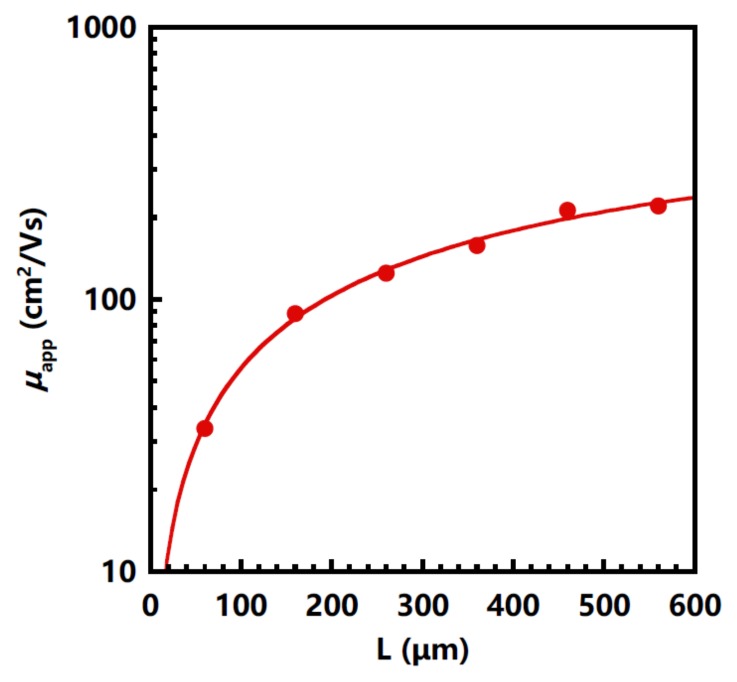
The extracted apparent field-effect mobility μ${}_{app}$ as a function of channel length and the fitting result considering the contact resistance.

Figure 3 shows the dependence characteristics of the channel length on the apparent field-effect mobility (μ${}_{app}$) of the a-IGZO TFTs. As the channel length decreased from 560 μm to 60 μm, the μ${}_{app}$ dropped from 220.1 cm${}^{2}$/Vs to 33.5 cm${}^{2}$/Vs. This decrease should be due to the reduction of the drain voltage drop as channel length reduces. Therefore, we used the following equation to exclude the influence of the contact resistance to extract the intrinsic mobility of the TFTs [15]:

$$\mu_{app}=\mu_{ins}\frac{1}{1+R_{C}\frac{W}{L}C_{i}\mu_{ins}\left(V_{G}-V_{T}\right)},$$

where μ${}_{ins}$ is the intrinsic mobility and μ${}_{app}$ is the apparent field-effect mobility extracted in the linear regime of $I_{D}$-$V_{G}$ curves at $V_{D}$ = 0.1 V. The equation was used to fit the experimental results in Figure 3. A good agreement between the experimental results and the fitting was achieved. The extracted value of the μ${}_{ins}$ was 675.8 cm${}^{2}$/Vs.

**Figure 4:**
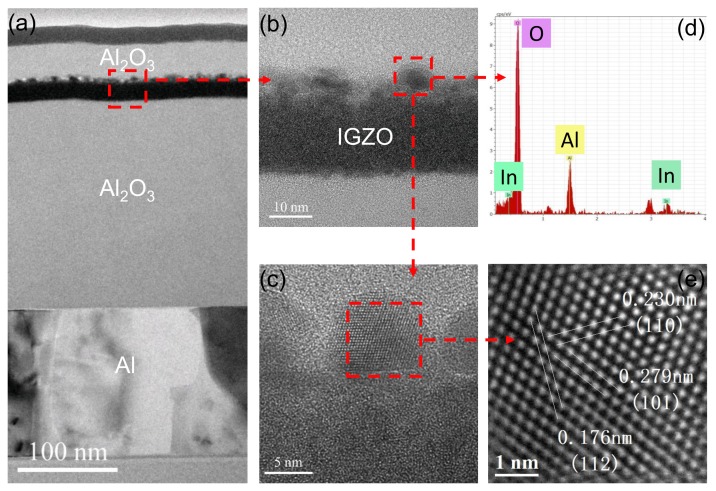
(**a**) cross-sectional image of a-IGZO TFT with Cu S/D and Al${}_{2}$O${}_{3}$ PVL; the (**b**) 450 k and (**c**) 910 k magnified image of the interface between a-IGZO and Al${}_{2}$O${}_{3}$ PVL; (**d**) corresponding EDS and (**e**) HR-TEM image of the particles.

High-resolution transmission electron microscopy (HR-TEM) integrated with X-ray energy dispersive spectroscopy (EDS) was performed to clarify the interface reaction of Al${}_{2}$O${}_{3}$ PVL with the a-IGZO channel layer. Figure 4a shows a cross-sectional HR-TEM image of the S5 device channel, while Figure 4b,c shows a magnified image taken from Figure 4a. The interfacial morphology between Al${}_{2}$O${}_{3}$ PVL and the a-IGZO film was clearly observed. We note that a thin layer formed at the interface was most likely induced by the sputtering of Al${}_{2}$O${}_{3}$ PVL. Figure 4d shows the corresponding chemical composition of the particle comprising In, Al and O. The HR-TEM image illustrated in Figure 4e indicated a single-crystalline feature where the internal plane spacing of 0.230 nm, 0.279 nm and 0.176 nm were indexed, which matched with the (110), (101) and (112) planes of metallic indium, respectively. Based on the above results, it is entirely reasonable to assume that the bombardment of energetic particles dissociates oxygen in indium oxide during the sputtering of Al${}_{2}$O${}_{3}$ PVL to form metallic indium nanoparticles. Moreover, these indium nanoparticles can increase carrier concentration and act as conduction paths to lower the channel resistance. However, as the carrier concentration in the channel increased, the depletion layer capacitance also increased, which resulted in a larger *SS* than the device without the Al${}_{2}$O${}_{3}$ PVL [16].

**Figure 5:**
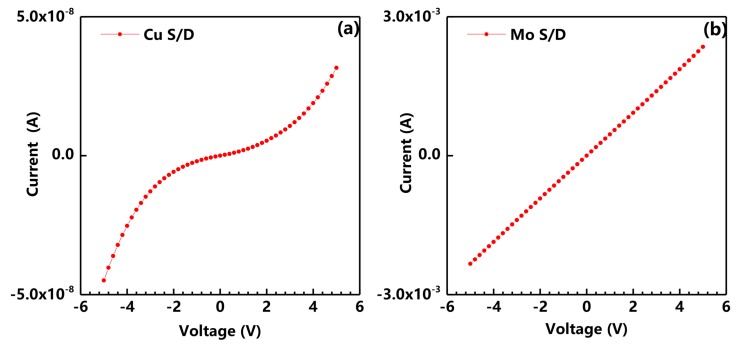
Current-Voltage (I-V) characteristics of a-IGZO film with different metal electrodes Cu S/D (**a**) and Mo S/D (**b**).

Current-voltage (I-V) characteristics of the a-IGZO films with different metal electrodes were used to investigate contact properties. Figure 5 shows the *I*-V characteristics of a-IGZO films with Cu and Mo S/D. As shown in the figure, the Mo-contact film showed perfect ohmic contact with linear curves. Moreover, the Cu-contact film exhibited regular Schottky contact. It is widely known that Cu doping is introduced as acceptors or acceptor-like traps in ZnO [17] and InGaZnO${}_{x}$ [18] films to suppress the carrier concentration because the chemical bonds of Cu-O form a covalent hybridized band between the O 2p and Cu 3d orbitals at the top of the valence band, resulting in p-type properties [19]. In this case, a decrease in the carrier concentration beneath the contact area due to Cu diffusion results in a high contact potential, which makes the Cu-contact device behave like a Schottky contact. From these results, the operating mechanism of the high mobility TFT can be attributed to the formation of the Schottky contact. As mentioned by previous researchers [20,21], the Schottky contact induces a high barrier at both ends of the S and D electrodes, which prevents the electrons from passing freely through the channel at a high negative gate voltage, thereby suppressing the leakage current of the TFT, even if the active layer is highly conductive. Thus, the TFT can be switched on/off normally.

**Figure 6:**
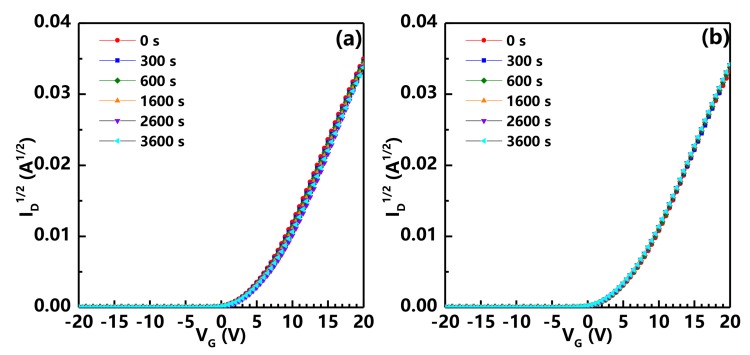
Variations of the transfer characteristics of a-IGZO TFT with Cu S/D and Al${}_{2}$O${}_{3}$ PVL after post-annealing under (**a**) positive and (**b**) negative bias stress for 3600 s.

The electrical stability of the S6 device under gate bias stress ($V_{G}$ = 10 V for positive bias stress (PBS) and –10 V for negative bias stress (NBS), $V_{D}$ fixed at 10 V) is also investigated, as shown in Figure 6. It is clearly observed that the TFT exhibits excellent electrical stability with the $V_{th}$ shift of only 0.6 V for PBS and −0.2 V for NBS, indicating that the Al${}_{2}$O${}_{3}$ PVL can effectively protect the TFT from the ambient influence.

## 3. Materials and Methods

A bottom-gate, top-contact configuration was used in the a-IGZO TFTs. Firstly, an Al gate electrode was deposited on the glass with a thickness of 300 nm. Then, an anodic Al${}_{2}$O${}_{3}$ gate insulator layer having a thickness of 190 nm was formed on the surface of the gate electrode by an anodic oxidation process. Details of the anodic oxidation technology have ever been reported elsewhere [22]. Then, a 25 nm thick a-IGZO (In:Ga:Zn = 1:1:1) film was deposited by radio frequency (RF) magnetron sputtering and patterned through a metal shadow mask. After that, the a-IGZO thin film was pre-annealed at 450 ${}^{\circ }$C for 1 h in the air. Cu or Mo thin films (200 nm of thickness), as source and drain electrodes (S/D), were deposited on an a-IGZO layer by direct current (DC) magnetron sputtering and patterned using a shadow mask. After that, a 30-nm thick Al${}_{2}$O${}_{3}$ thin film, as a passivation layer (PVL), was formed by RF magnetron sputtering using an Al${}_{2}$O${}_{3}$ target. Finally, a post-annealed treatment was carried out in an argon atmosphere at 350 ${}^{\circ }$C for 1 h.

The cross-sectional properties of each functional film were characterized by transmission electron microscopy (TEM, FEI HELIOS NANOLAB 450s, Hillsboro, OR, USA) equipped with an X-ray energy-dispersive spectroscopy (EDS). The electrical characterization was conducted by a semiconductor parameter analyzer (Agilent 4155C, Santa Clara, CA, USA) in the air.

## 4. Conclusions

In summary, we successfully fabricated a high-performance a-IGZO TFT based on Cu S/D with Al${}_{2}$O${}_{3}$ PVL. According to the TEM/EDS analyses, it is found that an In-rich layer formed after the sputtering of Al${}_{2}$O${}_{3}$ PVL. The conductive In nanoparticles in the In-rich layer serve as conduction paths to accelerate the carrier transport. Moreover, a Schottky contact created at the Cu S/D and a-IGZO channel interfaces due to Cu diffusion caused by the post-annealing treatment. Thus, the TFT with long channel length exhibited good switching operations and a high mobility exceeding 200 cm${}^{2}$/Vs. Moreover, the TFT also showed superior positive and negative gate bias stress stability.

## Figures and Tables

**Table 1:** Device performance for both a-IGZO TFTs.

Sample	S/D	Al${}_{2}$O${}_{3}$ PVL	Post-Annealing	*V*${}_{\mathrm{th}}$ (V)	μ${}_{\mathrm{FE}}$ (cm${}^{2}$/Vs)	*SS* (V/dec)	*I*${}_{\mathrm{on}}$/*I*${}_{\mathrm{off}}$
S1	Cu	No	No	3.5	8.2	0.26	3.3 × 10${}^{7}$
S2	Cu	No	Yes	10.0	6.8	0.43	9.2 × 10${}^{6}$
S3	Mo	Yes	No	—	—	—	—
S4	Cu	Yes	No	—	—	—	—
S5	Mo	Yes	Yes	—	—	—	—
S6	Cu	Yes	Yes	5.7	220.7	0.78	1.1 × 10${}^{8}$
